# Upon the Morbid Desire to Kill

**Published:** 1854-01-01

**Authors:** Raimundo D. Y. Correa


### UPON THE MORBID DESIRE TO KILL.

BY DON RAIMUNDO D. Y. CORREA.

[We select the following for several reasons—because it is by a Spaniard, and because it seems to us as bringing prominently forward several points in aid of the settlement (if ever it can be settled) of this question. Witness a late case, where the jury acquitted a seduced female of the crime of murder, on the score of insanity, and the next moment the judge gave her a free discharge, because he could see no proof of insanity. The article itself purports to be taken from the *Gac. De Madrid*, and may be found in the Medical Times and Gazette of January 29th, 1853, and we can hardly gather from it what portion of it belongs to the author or to the translator.—*American Journal of Insanity for Oct.*, 1853.]

The author commences with a eulogy on Esquirol's work on Homicidal Monomania, 1837, and quotes examples from Pinel, Marc, Gall, and Mende. These show the existence of a partial delirium, whether in the form of a fixed idea or an excited sensibility, extravagance in the passions, or error in judgment. In every instance there has been disturbance of the mind, and hence the words addressed by an advocate to Dr. Marc, upon the occasion of a trial of simple barbarity. "If monomania be a disease, its cure is upon the scaffold." The following bit of legal bloodthirstiness also merits being handed to posterity: "Your so-called homicidal monomania is an hypothesis, a modern and convenient invention to shield the guilty, and to withdraw them from the power of the law."

The author proceeds to say, that from the works of Magendie and other physiologists, he can prove that there are certain powers in man, which drive him in a definite direction, without his possessing a will sufficiently strong to offer opposition. These powers, which can be reduced to four, reside in the corpora striata, the cerebellum, the crura cerebri, and the medulla oblongata. Injuries to these parts in animals, cause different involuntary movements, and the author concludes therefrom that there are in man different impulses stronger than the will. Governed by these impulses, the homicidal maniac commits his crime.

A man who commits murder upon a false idea, with powerful impulse, should be considered as suffering from disease in the same part of the brain. Now we cannot see the application of Magendie or Flourens' experiments in the elucidation of psychical disturbance, nor comprehend why, upon division of the crura cerebri, the injured and dizzy animal rolls over and over. We have before complained with justice of the gross ignorance of morbid anatomy displayed by the generality of "mental physicians" in all countries, and we think it hard to refute statements made upon no foundation whatever. The examination of the bodies of criminal lunatics does not confirm in any one point the loose assertions of Dr. Raimundo. Neither the corpora striata nor the crura cerebri are often found in an abnormal condition; the cerebellum is for the most part natural in structure; the medulla oblongata unaltered, except that the membranes covering it become thickened, when other parts of the encephalic coverings have undergone a similar change. We have no ground whatever for asserting that these parts are essentially the seat of morbid actions during life. The cause of insanity is to be sought for in a source deeper that that supposed by those philosophers of a somewhat materialistic school. The author endeavours for judicial purposes to found a differential diagnosis between the maniac and the responsible culprit, both of whom have committed murder.

| Homicidal Monomania. | Criminal Murder. |
| --- | --- |
| The person is one of weak constitution, of nervous excitable temperament, irreproachable character, working in business for the immediate necessaries of life. | The criminals are mostly persons of strong constitution, sanguineous or choleric temperament, bad education, given to idle courses, and occupied in immoral pursuits. |
| The monomaniac is alone. | The criminal is rarely alone; has usually accomplices to share the booty. |
| The maniac kills without interest or passion, without motive, making that man an offering who may be unfortunate enough to meet him. | The criminal has a motive; has some passion to gratify, and selects his object accordingly. |
| The maniac disdains to fly, and often gives himself up to justice; he often details the particulars of his act, and seeks punishment more than pardon. | The criminal withdraws from observation; tries to mislead the judge; to cast suspicion on others, and to do his best to avoid punishment. |

We doubt if these aphorisms will stand their gound as unerring tests in this difficult question. The records of the criminal department of Bethlem Hospital would point to many an inmate imprisoned for murder, whose constitution was good and frame powerful and muscular. Many a criminal has had sufficient nerve to take life alone, unassisted by others; even the last who forfeited his life in the metropolis fell under this class. Should we be justified in asserting that he was mad, because he was *alone* in his wife's chamber when he cut her throat?

Again, the maniac mostly takes life, not by chance or hazard, but in obedience to a fixed, though erroneous idea, sometimes in sudden passion. Who can at all times either discover or appreciate motives?

Jealousy, hatred, or revenge, carefully guarded from public notice, would, but for the Law, impel many a ruffian to gratify his passion at the cost of another's life.

The maniac does not always disdain to fly, and can even argue cleverly in his own defence. But what can be said of that class of offenders whose lowly-organised and ill-directed minds are equally under the influence of both fear and evil passions? Place them under restraint, they behave respectfully and with decency, give them liberty and passion soon regains the mastery. Can any aphorisms comprehend the anomalies of this class? We believe not. Each case must be determined by circumstances elicited at the trial, and by the opinions of those in whom the responsibility of the judgment rests.

---

